# Blood Eosinophil Depletion with Mepolizumab, Benralizumab, and Prednisolone in Eosinophilic Asthma

**DOI:** 10.1164/rccm.202003-0729LE

**Published:** 2020-11-01

**Authors:** Angela M. Moran, Sanjay Ramakrishnan, Catherine A. Borg, Clare M. Connolly, Simon Couillard, Christine M. Mwasuku, Ian D. Pavord, Timothy S. C. Hinks, Lauri Lehtimӓki

**Affiliations:** ^1^ University of Oxford, Oxfordshire, United Kingdom; ^2^ Faculté de Médecine et des Sciences de la Santé de l’Université de Sherbrooke, Sherbrooke, Quebec, Canada and; ^3^ Tampere University Hospital, Tampere, Finland

*To the Editor*:

Eosinophilic airway inflammation is present in approximately half of patients with asthma, and it is particularly associated with asthma attacks. IL-5 is a major cytokine involved in eosinophil differentiation, proliferation, activation, and eosinophil-mediated inflammatory responses (1). Severe eosinophilic asthma can now be effectively treated using anti–IL-5 biologic therapies. The two anti–IL-5 monoclonal antibodies that are licensed for use in asthma and can be administered subcutaneously are mepolizumab, targeting IL-5, and benralizumab, targeting the α-chain of the IL-5 receptor. They both have proven efficacy in decreasing the frequency of asthma exacerbations and oral corticosteroid requirement (2–5). In preclinical studies, benralizumab has additional antibody-dependent cell-mediated cytotoxicity effects and might therefore deplete blood eosinophils more quickly and completely than mepolizumab (6).

Pharmacodynamic studies of mepolizumab and benralizumab have only been undertaken over longer time periods, and their findings are difficult to compare, as baseline and change in blood eosinophils have been expressed differently (7–9). There are no data comparing the effect of mepolizumab and benralizumab on blood eosinophils in the first 24 hours after administration. This is an important gap in knowledge, as rapid onset depletion of blood eosinophils is likely to be an important property if anti–IL-5 biologics are to be used as an alternative for prednisolone as a treatment of acute eosinophilic exacerbations (10). We hypothesized that benralizumab and prednisolone might work equally rapidly and faster than mepolizumab because of induction of apoptosis and antibody-dependent cell-mediated cytotoxicity. We have therefore undertaken a study to compare the rate of blood eosinophil depletion after administration of the first dose of mepolizumab, benralizumab, and prednisolone in patients with severe eosinophilic asthma.

## Methods

This was a substudy of the Oxford Airways Study (Integrated Research Application System Project number: 234581; Oxfordshire Research Ethics Committee B Reference: 18/SC/0361). The Oxford Airways Study is an observational study of patients with asthma and chronic obstructive pulmonary disease, which allows blood, sputum, and bronchoscopy sampling at stable state and during exacerbations, before and after clinical treatment. The main outcome of this substudy was time to reach a 50% reduction in blood eosinophil count after administration of mepolizumab, benralizumab, and prednisolone. Patients were recruited from the Oxford Special Airways Clinic. Inclusion criteria were having a known diagnosis of severe asthma and baseline blood eosinophil count above 300 cells/μl. Subjects who had taken prednisolone in the preceding 2 weeks or who were already on a biologic treatment were excluded. We recruited 18 patients who were either commencing prednisolone 30 mg daily for 5 days (*n* = 6) for the treatment of poorly controlled eosinophilic asthma or commencing maintenance treatment with mepolizumab 100 mg subcutaneously (*n* = 6) or benralizumab 30 mg subcutaneously (*n* = 6) for poorly controlled severe eosinophilic asthma (5.4 subjects in each group is needed for a statistical power of 90% [α = 0.05] to detect a between-group difference larger than 1 SD). We assessed blood eosinophil counts at baseline (generally 9 a.m.), at 2 and 4 hours after the first dose for all patients, and at 6, 8, 24, and 96 hours if the blood eosinophil count remained higher than 150 cells/μl. A 30-day blood eosinophil count was obtained for those who received the mepolizumab or benralizumab injection. There is no previous information on the onset of action of mepolizumab and benralizumab; however, the effect of prednisolone starts in about 2 hours, so we included the time points 2 and 4 hours, and later time points to show the predicted slower onset of mepolizumab. Hematological analysis was done by laboratory staff using an Abbott Architect ci8200 analyzer.

## Results

**Figure 1.**
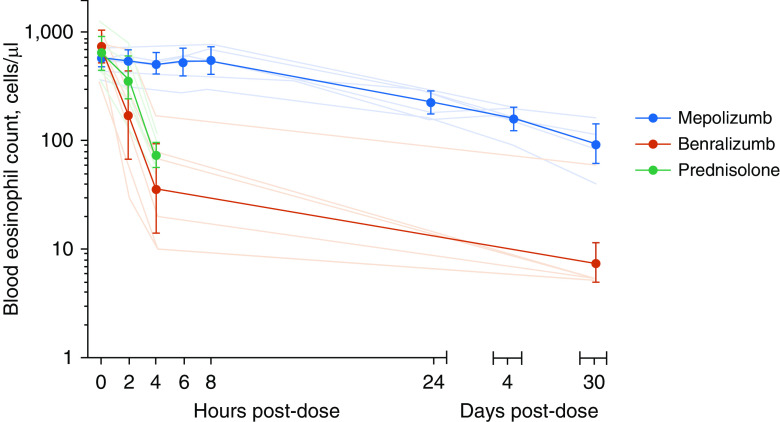
Blood eosinophil count (thin transparent lines for individual data, thick lines with markers for geometric group mean, and error bars represent 95% confidence intervals for geometric mean) on logarithmic scale before and after treatment with mepolizumab (100 mg s.c.), benralizumab (30 mg s.c.), or oral prednisolone (30 mg). There was a statistically significant change (*P* < 0.05 using repeated-measures ANOVA and least significant difference *post hoc* test) from baseline at 2 and 4 hours in the benralizumab arm, at 4 hours in the prednisolone arm, and from 24 hours onward in the mepolizumab arm. There was no significant difference between the treatment arms at baseline, but the mepolizumab arm differed significantly from benralizumab arm at 2 and 4 hours and 30 days and from prednisolone arm at 4 hours (ANOVA with least significant difference *post hoc* test).

Demographics, inhaled corticosteroid dose, lung function, baseline blood eosinophil count, and exhaled nitric oxide levels were not significantly different between groups (Table 1). The mean (SD) time for blood eosinophil level to decrease 50% from baseline was 25.8 (14.3) hours on mepolizumab, 1.7 (0.7) hours on benralizumab, and 2.5 (0.3) hours on prednisolone (*P* < 0.001 for both benralizumab and prednisolone compared with mepolizumab, and *P* = 0.874 between prednisolone and benralizumab by one way ANOVA with least significant difference *post hoc* test) (Figure 1). A blood eosinophil count ≤100 cells/μl was achieved by one patient treated with mepolizumab at 96 hours and by three patients at 30 days; this threshold was achieved by 4 hours in five participants treated with benralizumab and five treated with prednisolone. Blood eosinophil count in the mepolizumab arm was significantly higher than in the benralizumab arm at 2 and 4 hours and 30 days (*P* = 0.045, <0.001, and 0.002, respectively) and higher than in the prednisolone arm at 4 hours (*P* < 0.001, Figure 1). There was no statistically significant difference between the prednisolone and benralizumab arms at any of the time points (*P* = 0.601 at 0 hours, *P* = 0.296 at 2 hours, and *P* = 0.767 at 4 hours). The geometric mean (geometric SD) blood eosinophil counts 30 days after benralizumab and mepolizumab were 8 (2.8) and 92 (1.7) cells/μl, respectively (*P* = 0.002).

**Table 1.** Clinical Characteristics of Participants

	Mepolizumab	Benralizumab	Prednisolone	*P* Value
n	6	6	6	—
Age, yr*	57 (53–64)	68 (54–69)	53 (46–67)	0.5
Females	3 (50)	2 (33)	1 (17)	0.8
Ethnicity				
Caucasian	6 (100)	5 (83)	6 (100)	0.3
African	—	1 (17)	—	—
Sensitization to any respiratory allergens	3 (50)	4 (67)	2 (33)	0.5
ICS BDP equivalent dose, mcg/d*	2,000 (1,900–3,100)	2,000 (1,750–2,000)	2,000 (1,900–2,000)	0.1
No. OCS courses past 12 mo*	8 (5–11)	4 (3–6)	3 (2–5)	0.2
FEV_1_% predicted*	68 (37–78)	71 (57–98)	73 (57–87)	0.4
FVC % predicted*	75 (69–89)	87 (70–128)	88 (73–96)	0.4
FEV_1_/FVC ratio*	0.66 (0.41–0.84)	0.71 (0.39–0.74)	0.75 (0.57–0.81)	0.4
Blood eosinophil count, cells/μl^†^	580 (1.3)	724 (1.5)	629 (1.6)	0.5
Fe_NO_, ppb*	41 (16–97)	61 (33–130)	50 (20–81)	0.4

*Definition of abbreviations*: BDP = beclomethasone dipropionate; ICS = inhaled corticosteroids; OCS = oral corticosteroids; Fe_NO_ = fractional exhaled nitric oxide.

*P* values are for chi-test, Kruskall-Wallis test, or ANOVA. Results are expressed as number (percentage of the group) unless otherwise specified.

* Median (interquartile range).

^†^ Geometric mean (geometric SD).

## Discussion

To the best of our knowledge, this is the first study comparing the rate of depletion of blood eosinophils in the first 24 hours after treatment with mepolizumab and benralizumab. We found that, in contrast to mepolizumab, benralizumab caused rapid and near complete depletion of eosinophils with a speed of onset of effect very similar to that seen with oral prednisolone. The blood eosinophil count 30 days after the first injection was also significantly lower after benralizumab compared with mepolizumab. These findings are consistent with greater efficacy and a different mechanism of eosinophil depletion.

Mepolizumab and benralizumab have proven and similar efficacy and safety in the longer-term treatment of severe eosinophilic asthma (2, 3), suggesting that the difference in the speed of onset and efficacy is not relevant to the chronic use of these agents. However, the faster onset of action of benralizumab suggests it has potential as an alternative noncorticosteroid treatment for acute exacerbations of eosinophilic asthma with the potential benefit of inhibiting eosinophilic airway inflammation for at least 30 days after one injection. A recent case report supports the use of benralizumab in this way (10). Larger studies are needed to determine whether this strategy is clinically effective and cost effective.

## Supplementary Material

Supplements

Author disclosures

## References

[1] Lambrecht BN Hammad H The immunology of asthma *Nat Immunol* 2015 16 45 56 25521684 10.1038/ni.3049

[2] Pavord ID Korn S Howarth P Bleecker ER Buhl R Keene ON *et al* Mepolizumab for severe eosinophilic asthma (DREAM): a multicentre, double-blind, placebo-controlled trial *Lancet* 2012 380 651 659 22901886 10.1016/S0140-6736(12)60988-X

[3] Bleecker ER FitzGerald JM Chanez P Papi A Weinstein SF Barker P *et al* SIROCCO Study Investigators Efficacy and safety of benralizumab for patients with severe asthma uncontrolled with high-dosage inhaled corticosteroids and long-acting β_2_-agonists (SIROCCO): a randomised, multicentre, placebo-controlled phase 3 trial *Lancet* 2016 388 2115 2127 27609408 10.1016/S0140-6736(16)31324-1

[4] Nair P Wenzel S Rabe KF Bourdin A Lugogo NL Kuna P *et al* ZONDA Trial Investigators Oral glucocorticoid-sparing effect of benralizumab in severe asthma *N Engl J Med* 2017 376 2448 2458 28530840 10.1056/NEJMoa1703501

[5] Bel EH Wenzel SE Thompson PJ Prazma CM Keene ON Yancey SW *et al* SIRIUS Investigators Oral glucocorticoid-sparing effect of mepolizumab in eosinophilic asthma *N Engl J Med* 2014 371 1189 1197 25199060 10.1056/NEJMoa1403291

[6] Kolbeck R Kozhich A Koike M Peng L Andersson CK Damschroder MM *et al* MEDI-563, a humanized anti-IL-5 receptor alpha mAb with enhanced antibody-dependent cell-mediated cytotoxicity function *J Allergy Clin Immunol* 2010 125 1344 1353, e2 20513525 10.1016/j.jaci.2010.04.004

[7] Wang B Yan L Yao Z Roskos LK Population pharmacokinetics and pharmacodynamics of benralizumab in healthy volunteers and patients with asthma *CPT Pharmacometrics Syst Pharmacol* 2017 6 249 257 28109128 10.1002/psp4.12160 PMC5397562

[8] Smith DA Minthorn EA Beerahee M Pharmacokinetics and pharmacodynamics of mepolizumab, an anti-interleukin-5 monoclonal antibody *Clin Pharmacokinet* 2011 50 215 227 21348536 10.2165/11584340-000000000-00000

[9] Leckie MJ ten Brinke A Khan J Diamant Z O’Connor BJ Walls CM *et al* Effects of an interleukin-5 blocking monoclonal antibody on eosinophils, airway hyper-responsiveness, and the late asthmatic response *Lancet* 2000 356 2144 2148 11191542 10.1016/s0140-6736(00)03496-6

[10] Ramakrishnan S Camp JR Vijayakumar B Hardinge FM Downs ML Russell REK *et al* The use of benralizumab in the treatment of near-fatal asthma: a new approach *Am J Respir Crit Care Med* 2020 201 1441 1443 32023077 10.1164/rccm.202001-0093LE PMC7258629

