# Supplementary material for: Blood Eosinophil Depletion with Mepolizumab, Benralizumab, and Prednisolone in Eosinophilic Asthma
Source: Am J Respir Crit Care Med. 2020 Nov 1;202(9):1314–6. doi: 10.1164/rccm.202003-0729LE (PMC7605198; doi:10.1164/rccm.202003-0729LE)
Supplement: Supplements [file rccm.202003-0729LE.html]

Blood Eosinophil Depletion with Mepolizumab, Benralizumab, and Prednisolone in Eosinophilic Asthma | American Journal of Respiratory and Critical Care Medicine

- disclosures.pdf (249 KB)
